# Anti-arrhythmic effect of acupuncture pretreatment in the rats subjected to simulative global ischemia and reperfusion—involvement of intracellular Ca^2+^ and connexin 43

**DOI:** 10.1186/s12906-015-0521-y

**Published:** 2015-02-05

**Authors:** Junhong Gao, Yuxue Zhao, Yumin Wang, Juanjuan Xin, Jingjing Cui, Shuhua Ma, Fengyan Lu, Lianping Qin, Xiaochun Yu

**Affiliations:** Department of Physiology, Institute of Acupuncture, China Academy of Chinese Medical Sciences, No. 16 Nanxiaojie, Dongcheng District, Beijing, 100700 Dongzhimennei China; Department of Oncology, The Affiliated Hospital of Chifeng University, Chifeng, 024005 Inner Mongolia China; Department of Physiology, The Experimental Research Center, China Academy of Chinese Medical Sciences, No. 16 Nanxiaojie, Dongcheng District, Beijing, 100700 Dongzhimennei China

**Keywords:** Electro-acupuncture, Pretreatment, Arrhythmia, Intracellular Ca^2+^, Connexin 43

## Abstract

**Background:**

The previous study showed that the cardiac arrhythmias induced by myocardial ischemia and reperfusion were attenuated by the pretreatment of acupuncture; however, the related mechanism is not understood. The present study was therefore designed to determine whether intracellular Ca^2+^ ([Ca^2+^]_i_) and connexin 43 (Cx_43_) are involved in the mediation of the anti-arrhythmic effect of electro-acupuncture (EA) pretreatment in the rats subjected to simulative global ischemia and reperfusion (SGIR).

**Methods:**

SGIR was made in the isolated heart by a low flow perfusion followed by a flow restoration. Four groups of animals are involved in the present study, including normal control group, SGIR group, EA group and EA plus 18 beta-glycyrrhetinic acid (EAG) group. For EA pretreatment, bilateral Neiguan acupoints (PC6) of the rats were stimulated for 30 min once a day in 3 consecutive days. Cx_43_ antagonist was given to the rats in EAG group 30 minutes before the EA pretreatment. The resting [Ca^2+^]_i_ concentration, calcium oscillation, the contents of total Cx_43_ and non-phosphrylated Cx_43_ and arrhythmia score were compared among different groups.

**Results:**

In EA group, the arrhythmic score, the resting [Ca^2+^]_i_ concentration and the number of [Ca^2+^]_i_ oscillations were all significantly less than those in SGIR group (all *P* < 0.05), and interestingly, after EA pretreatment, the contents of nonphosphated Cx43 in the EA group were significantly lower than that in SGIR group respectively (*P* < 0.05). However, when the rats were treated with Cx_43_ antagonist prior to the EA pretreatment, the protection effects induced by EA pretreatment were reversed.

**Conclusions:**

The results showed that EA pretreatment could produce anti-arrhythmic effect in the rats subjected to SGIR. The anti-arrhythmic effect of EA pretreatment may be due at least partially to the inhibition of SGIR-induced calcium overload and [Ca^2+^]_i_ oscillations, reduction of non-phosphorylated Cx_43_ and the enhancement of the corresponding phosphorylated Cx_43_ in the cardiac cells.

**Electronic supplementary material:**

The online version of this article (doi:10.1186/s12906-015-0521-y) contains supplementary material, which is available to authorized users.

## Background

The sudden cardiac death can be easily induced by the severe ischemic arrhythmias including ventricular fibrillation occurred during the myocardial ischemia [1]. Acupuncture was reported to improve myocardial ischemia and attenuate arrhythmias [2-4]^.^ In early 1980s, Guo and colleagues reported that the extrasystole induced by hypothalamic stimulation could be inhibited significantly by the somatic nerve stimulation [5,6]. More recently, the electro-acupuncture (EA) was shown to diminish the susceptibility to ventricular tachycardia by reducing cardiac metabolic demand [7]. A possible important role of intracellular Ca^2+^ ([Ca^2+^]_i_) in the mediation of the anti-arrhythmic effect of EA has been suggested by Longurst [3]. Our previous study showed that the cardiac arrhythmias induced by myocardial ischemia and reperfusion (MIR) could be attenuated by the pretreatment of acupuncture via inhibiting the ischemia-elevated response of [Ca^2+^]_i_ [8]. However, detailed mechanisms underlying the anti-arrhythmic effect of acupuncture have not been elucidated yet.

The gap junction between the cardiac cells is composed of two connexins from the neighboring cardiomyocytes’ membrane and plays an important role in the cell-to-cell communication between cardiac cells. In ventricle the major junction protein is connexin 43 (Cx_43_) which ensures cardiac electric conduction and electric synchronicity. Alteration of Cx_43_ in quantity, phosphorylation and distribution may cause cardiac electrically conductive disorder and result in arrhythmias eventually. Cx_43_ is known to be a functionally calcium-associated protein [9]. The previous experimental study has demonstrated that Cx_43_ remodeling may account for intercellular calcium overload which is also associated with induction of ischemic arrhythmia [10]. Actually, intracellular calcium oscillations, known to be related to the early and delayed afterdepolarizations, are directly associated with the occurrence of arrhythmias [11,12]. In addition, the arrhythmia-related calcium oscillations are frequently observed during MIR [13,14].

In order to determine whether Cx_43_ and [Ca^2+^]_i_ are involved in the mediation of the anti-arrhythmic effect produced by EA pretreatment, the arrhythmia score, resting [Ca^2+^]_i_ concentration, calcium oscillations, the content of total Cx_43_ protein and the content of non-phosphorylated Cx_43_ in cardiac myocytes isolated from the heart subjected to the simulative global ischemia-reperfusion (SGIR) were measured in the present study.

## Methods

### Animal grouping and electro-acupuncture pretreatment

The present study was approved by the Committee on the Use of Live Animals in Research of the China Academy of Chinese Medical Sciences. Male Sprague–Dawley rats weighing 200–225 g were randomly divided into four groups, namely, normal control (NC) group, simulative global ischemia-reperfusion (SGIR) group, electro-acupuncture (EA) group and EA plus 18 beta-glycyrrhetinic acid (EAG) group. Before the experiments, the animals in EA and EAG groups were both pretreated with EA applied at bilateral Neiguan acupoints (PC 6, according to the textbook of experimental acupuncture, Neiguan acupoint is located on forelimbs and was most frequently and effectively used to treat cardiac malfunctions including arrhythmias in clinical and experimental researches) under anesthesia with urethane (1 g/kg) for 30 min once a day for three consecutive days. For acupuncture manipulation, two needles, with 2–3 mm apart from each other, were inserted through the skin to a depth of about 2 mm at each Neiguan acupoint. And then they were connected to positive and negative poles of EA apparatus. The stimulatory intensity and frequency of EA were 1–3 mA and 20 Hz respectively. 18 beta-glycyrrhetinic acid, an antagonist of Cx_43_, was administered intraperitoneally in the rats of EAG group at a dose of 160ug/kg 30 min before EA pretreatment. The animals in NC and SGIR groups were treated equally as those in EA and EAG groups except the treatment of EA or 18 beta-glycyrrhetinic acid.

### Langendorff perfused isolated rat heart preparation

The Langendorff isolated perfused rat hearts were prepared for the study of arrhythmias as described previously [15]. In brief, male Sprague Dawley rats were sacrificed by decapitation with a guillotine immediately after the 3rd-day EA treatment was completed and arrhythmias recording were finished. Hearts were removed immediately and perfused retrogradely with a Krebs-Ringer solution which was aerated with 95% O_2_ and 5% CO_2_, pH 7.4, under a pressure of 55–70 mmHg and a constant flow rate of 13 ml/min. The temperature of the heart was maintained at 37°C. The first 10 min of perfusion allowed the heart to stabilize and any heart exhibiting arrhythmias during this period was discarded. The hearts in SGIR group, EA group and EAG group were initially perfused at a rate of 13 ml/min for 10 min, which was followed by a reduced-flow perfusion at a rate of 0.5 ml/min for 40 min. After reduced-flow perfusion, the perfusing flow was restored to the control level for 10 min. In NC group the hearts were perfused at a constant flow rate of 13 ml/min without reduced-flow perfusion for 60 min. Although there were occasional arrhythmias during the low flow period, arrhythmias appeared more frequently when the flow was restored as observed in previous study and our preliminary observation [16]. In the present study we determined the arrhythmias within 10 min immediately after the restoration of perfusion, namely, the reperfusion.

### ECG recording and arrhythmia scoring

ECG was continuously monitored with standard lead II throughout the experiment with a positive electrode hooked to the apex of the heart and a negative electrode at the aorta. To make comparison the arrhythmia scoring system modified from previous studies was adopted [17]. The principles of the scoring system employed were as follows. (1) Ventricular arrhythmias are more severe than atrial arrhythmias; (2) the severity of ventricular arrhythmias are ventricular fibrillation (VF), ventricular tachycardia (VT), frequent premature ventricular contraction (PVC) and occasional PVC in descending order; (3) the longer the duration of arrhythmias or the more frequent the incidence of arrhythmias, the greater the severity of arrhythmias. In the present study, the score of a heart was that of the most severe type of arrhythmia exhibited by the heart. The details of the scoring system are shown in the Table 1.

**Table 1:** **Arrhythmia scoring system**

**Arrhythmia score**	**Type of arrhythmia**
0	No arrhythmia
1	Atrial arrhythmias or occasional PVC
2	Frequent PVC
3	VT (1–2 episodes)
4	VT (>3 episodes) or VF (1–2 episodes)

### Measurement of cytosolic free [Ca^2+^]_i_

Ventricular myocytes of Sprague Dawley rats were isolated using a collagenase perfusion method [18]. The cells were loaded with fura-2/AM as a Ca^2+^ indicator, and [Ca^2+^]_i_ transient was determined by a spectrofluorometric method as described previously [19]. Briefly, the ventricular myocytes loaded with fura-2/AM were transferred to the stage of an inverted microscope (Nikon) in a superfusion chamber at room temperature. The ventricular myocytes were adapted for 20 min in the perfusion chamber before Ca^2+^ measurement. The fluctuation of [Ca^2+^]_i_ concentration was then recorded for 10 minutes in each selective resting single ventricular myocyte and the recorded values were averaged. A spike-like spontaneous increase in [Ca^2+^]_i_ with amplitude over two times of the standard deviation of the quiescent [Ca^2+^]_i_ variations was determined as an oscillation [20].

### Measurement of content of cardiac Cx_43_

Ventricular tissue of the heart was isolated in the end of perfusion experiments and immediately frozen. On the day of preparation of ventricular sarcolemma, the stored ventricle was homogenized in hypotonic membrane buffer containing 1 mM 1, 10-phenanthroline, 1 mM iodoacetamide, 1 mM pepstatin A, 0.4 mM phenylmethylsulfonyl fluoride (PMSF) with ultrasound homogenizer (Sonics & Material Inc, Jencons Ltd. Germany) . A modified Western blotting technique was used to determine the content of cardiac Cx_43_ [21].

### Drugs and chemicals

Type I collagenase, Joklik modified Eagle medium, HEPES, fura-2/AM, 18 and beta-glycyrrhetinic acid were purchased from Sigma Chemical Co; anti-glyceraldehyde-3-phosphate dehydrogenase antibody, polyclonal and monoclonal anti-Cx_43_ antibodies as well as the corresponding horseradish peroxidase-conjugated secondary antibodies were obtained from Santa Cruz Biotech. (Santa Cruz, CA). All the chemicals were dissolved in distilled water except fura-2/AM, which was dissolved in diethyl sulphoxide (DMSO).

### Statistical analysis

All the data in the present study are presented as Mean ± SE. A one-way ANOVA was used for multiple group comparisons and Dunnett’s *T*-test for two group comparison. *P* value less than 0.05 was considered as statistical significance.

## Results

### EA pretreatment and anti-arrhythmia in rats subjected to SGIR

**Figure 1:**
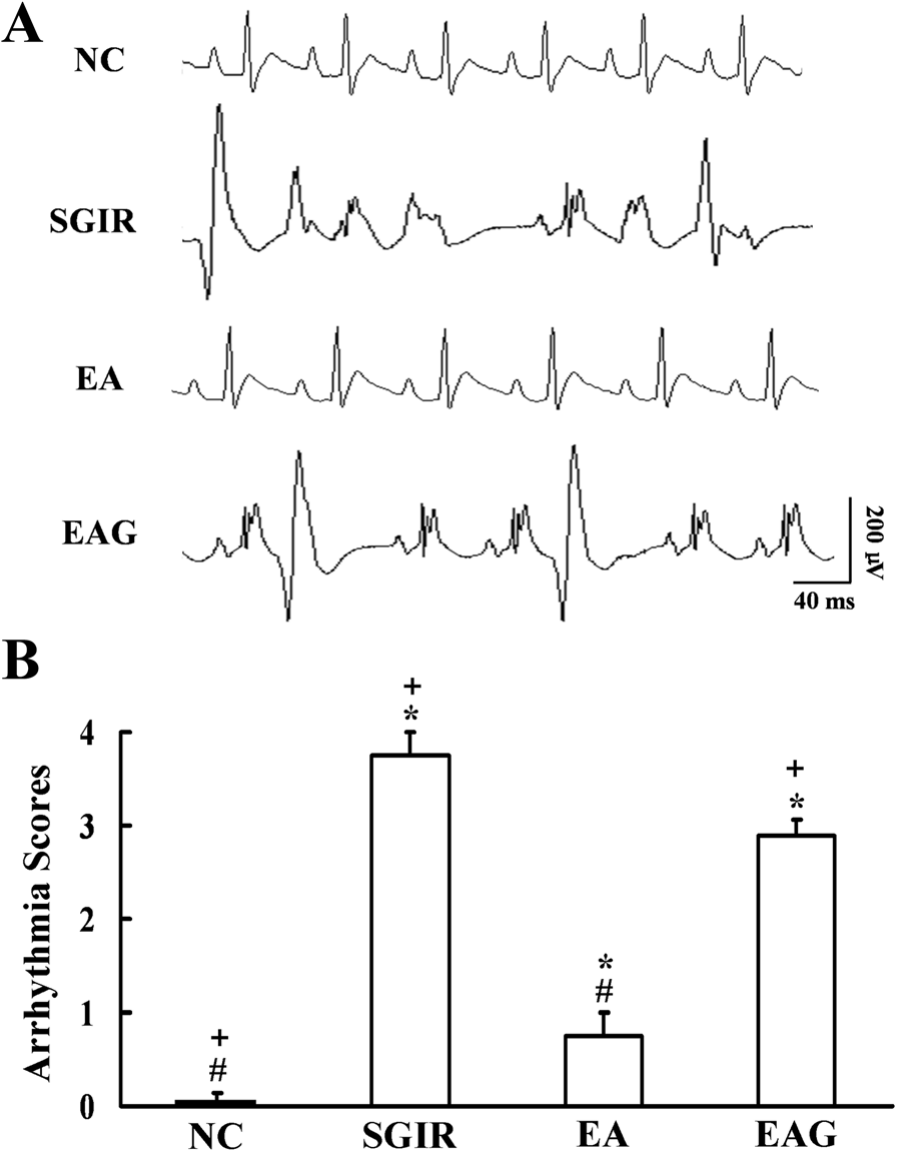
**Effect of EA pretreatment on ischemic arrythmias in the isolated heart subjected to SGIR.** Panel **A**: Representative traces of ECG showing the cardiac arrhythmias in the rats of different groups. Panel **B**: Statistical results of arrhythmic scores evaluating the cardiac arrhythmias recorded 10 minutes after reperfusion in the different groups. NC = normal control group; SGIR = simulative global ischemia & reperfusion group; EA = electro-acupuncture group; EAG = electro-acupuncture plus 18-beta-glycyrrhetinic acid group. *P < 0.05 vs. control group; #*P* < 0.05 as compared with SGIR group; +*P* < 0.05 in comparison with EA group. (n = 10).

Figure 1A shows the ECG traces recorded in isolated hearts from different groups. Arrhythmias were frequently observed in SGIR and EAG groups. The arrhythmic score (Figure 1B) was zero in NC group, suggesting no arrhythmia was observed. However, atrial and ventricular arrhythmias occurred in SGIR group with an arrhythmia score of 3.75 ± 0.25 (*P* < 0.05 vs NC group). Interestingly, the arrhythmia score was 0.75 ± 0.25 in EA group, indicating that the incidence of arrhythmias is significantly decreased (*P* < 0.05) with EA pretreatment. When the animals was pretreated intraperitoneally with the Cx_43_ inhibitor 18 beta-glycyrrhetinic acid, the arrhythmic score was 2.89 ± 0.18 with EA treatment. This result demonstrates Cx_43_ mediates the attenuation of arrhythmias induced by EA.

### EA pretreatment and [Ca^2+^]_i_ in resting single ventricular myocyte isolated from the perfused heart subjected to SGIR

**Figure 2:**
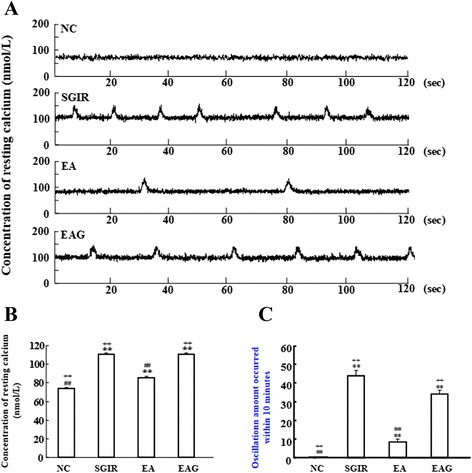
**Effect of EA pretreatment on [Ca** ^**2+**^ **]**_**i**_ **concentration and [Ca** ^**2+**^ **]**_**i**_ **oscillations in resting single ventricular myocyte.** Panel **A**: Representative traces of Ca^2+^ transient in resting single ventricular myocyte of different groups. Panel **B**: Statistical results of resting [Ca^2+^]_i_ concentration in single ventricular myocyte of different groups. Panel **C**: Statistical graph showing the oscillations in resting single ventricular myocyte of different groups. NC = normal control group; SGIR = simulative global ischemia & reperfusion group; EA = electro-acupuncture group; EAG = electro-acupuncture plus 18-beta-glycyrrhetinic acid group. **P < 0.01 vs. control group; ##*P* < 0.01 as compared with SGIR group; ++*P* < 0.01 in comparison with EA group. (n = 20).

Figure 2A displays the representative resting [Ca^2+^]_i_ recordings in ventricular myocytes. Spontaneous [Ca^2+^]_i_ oscillations were observed in the cells of SGIR group, but not in the cells of normal control group. The frequency of spontaneous [Ca^2+^]_i_ was reduced in the cells of EA group. Figure 2B illustrates the mean values of resting [Ca^2+^]_i_ and [Ca^2+^]_i_ oscillations in single ventricular myocyte from different groups. The resting [Ca^2+^]_i_ was 73.67 ± 1.08 nmol/L in NC group, 110.42 ± 1.49 nmol/L in SGIR group, 85.42 ± 1.56 nmol /L in EA group, and110.42 ± 1.64 nmol/L in EAG group. The mean value of resting [Ca^2+^]_i_ was significantly increased by SGIR (*P* < 0.01 vs NC group), and the increased resting [Ca^2+^]_i_ was remarkably countered by repetitive EA pretreatment (*P* < 0.01 vs SGIR group). Interestingly, the response of resting [Ca^2+^]_i_ to EA treatment was attenuated by the Cx_43_ inhibitor 18-beta-glycyrrhetinic acid (*P* < 0.01 vs EA group).

### EA pretreatment and SGIR-induced [Ca^2+^]_i_ oscillations in resting single ventricular myocyte

Figure 2C shows that no [Ca^2+^]_i_ oscillations in resting single ventricular myocyte from hearts of different groups. The [Ca^2+^]_i_ oscillation number in SGIR group was 43.83 ± 2.68, significantly higher than zero in NC group (*P* < 0.01); The increased [Ca^2+^]_i_ oscillation number was significantly reduced to 8.33 ± 1.58 in EA group (*P* < 0.01 vs SGIR group). The EA-reduced resting calcium oscillation number was reversed to 34.00 ± 2.11 by the Cx_43_ inhibitor 18-beta-glycyrrhetinic acid (*P* <0.01 vs EA group).

### EA pretreatment and Cx_43_ protein levels in ventricular myocytes

**Figure 3:**
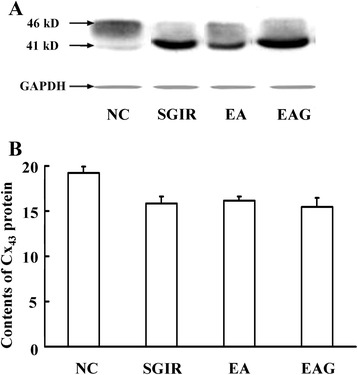
**Effect of EA pretreatment on Cx**_**43**_ **protein content in heart of rats subjected to SGIR.** Panel **A**: Representative traces of electrophoresis of myocardial Cx_43_ protein at 41 and 46 kilo-Dalton after probed with polyclonal anti-Cx_43_ antibody in different groups. Panel **B**: Group results showing the changes of relative density of myocardial Cx_43_ protein in different groups. Glyceraldehyde-3-phosphate dehydrogenase (GAPDH) was taken as the internal control. (n = 20).

Figure 3 displays the Western blot results for total Cx_43_ protein expression in the ventricular tissue from different groups. The total Cx_43_ (kD 46 and kD 41) was reduced by the different treatments of SGIR, EA and EAG. The average values of the optical density for Cx_43_ protein (relative to GAPDH) were 15.81 ± 0.79, 16.14 ± 0.44 and 15.48 ± 0.96 in SGIR, EA and EAG groups respectively, significantly lower than that (19.22 ± 0.69) in NC group. However, no significant difference was observed among SGIR, EA and EAG groups (*P* > 0.05).

### EA pretreatment and nonphosphorylated Cx_43_ in ventricular myocytes isolated from the perfused hearts subjected to SGIR

**Figure 4:**
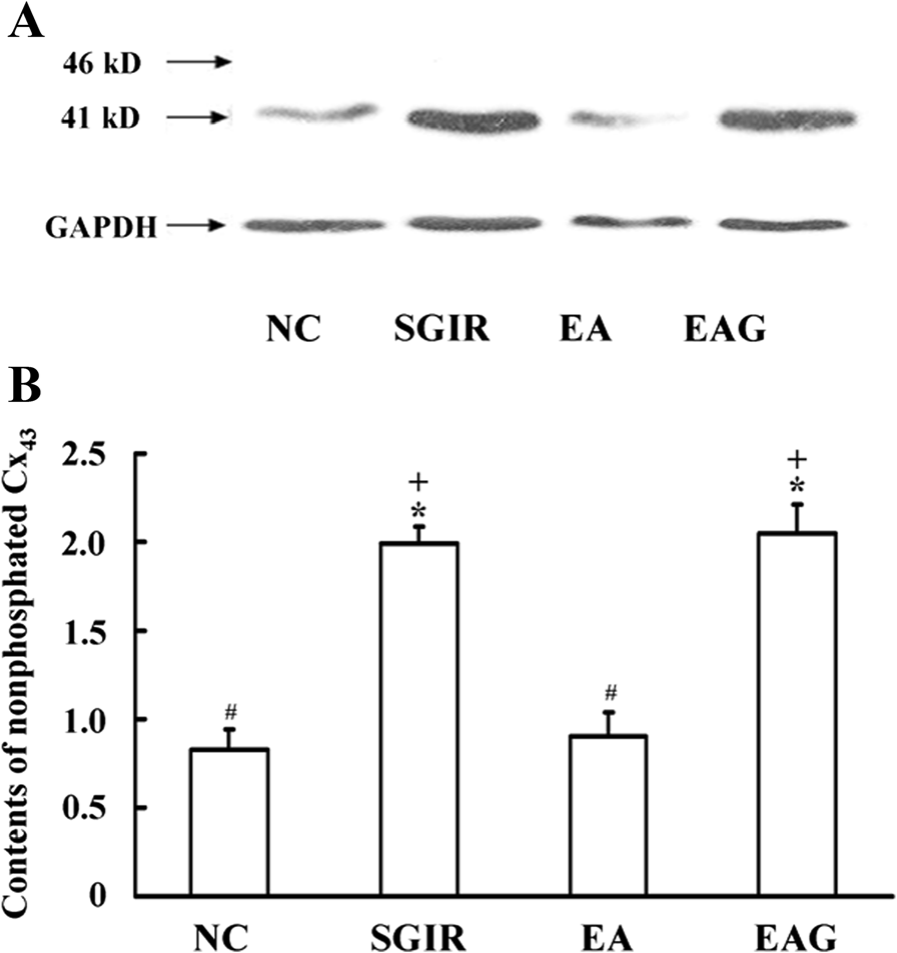
**Effect of EA pretreatment on Cx43 phosphorylation in perfused hearts subjected to SGIR.** Panel **A**: Representative traces of electrophoresis of myocardial Cx_43_ protein at 41 kilo-dalton after probed with monoclonal anti-Cx_43_ antibody in different groups. Panel **B**: Group results showing the changes of relative density of myocardial nonphosphorylated Cx_43_ protein in different groups. Glyceraldehyde-3-phosphate dehydrogenase (GAPDH) was taken as the internal control. *P < 0.05 as compared with control group; #*P* < 0.05 in comparison with SGIR group; +*P* < 0.05 in comparison with EA group (n = 20).

Figure 4 shows the optical protein density of nonphosphated Cx_43_ in different groups. The relative mean values of nonphosphated Cx_43_ level (Figure 4B) was increased to 1.99 ± 0.10 in SGIR group from 0.83 ± 0.11 of NC group (*P* < 0.05). The increased nonphosphated Cx_43_ level was significantly reduced to 0.90 ± 0.14 by EA pretreatment (*P* < 0.05 vs SGIR group); however, the EA effect was antagonized by the Cx_43_ inhibitor 18-beta-glycyrrhetinic acid (2.05 ± 0.16, *P* < 0.05 vs EA group).

## Discussion

Actually, acupuncture has been using by Chinese people for health care and prevention of various diseases since ancient time. Currently, acupuncture in China is being used not only to routinely treat patients with various diseases, but also to provide people with health care services like keeping fit, preventing aging, treating sub-health and so on.

In the past several decades scientists and medical doctors have tried their best to seek the preventive measures for the prevention of the acute attack of myocardial ischemia or coronary heart disease (CHD). It was found that ischemia preconditioning can effectively attenuate the cardiac injury induced by the subsequent severe myocardial ischemia. Unfortunately, this kind of protective or preventive measure is not clinically practicable. Acupuncture has been shown to attenuate the cardiac injury induced by MIR [22, 23]. However, patients with CHD rarely go to an acupuncturist because CHD at an acute stage is usually too urgent to be treated only by acupuncture. Therefore, seeking a proper way for acupuncture, an economic and simple therapy, to treat or prevent CHD is clinically beneficial. Pre-treatment with acupuncture, i.e., treating the people susceptible to CHD before they are acutely attacked, may be practicable in clinic for acupuncture to treat and prevent the disease.

The first observation in the present study is that the arrhythmic score in EA group was significantly less than that in SGIR group, indicating that an anti-arrhythmic effect is achieved by EA pretreatment. The results are similar to that of our previous study showing that the repetitive pretreatment with EA at PC6 acupoints produced a greater cardioprotective effect than the same stimulation at non-acupoints did [8].

As we know, in many clinical studies of acupuncture people have tried their best to design clinical trials carefully to eliminate the mental influence on the therapeutic effect of acupuncture. Therefore, although anesthesia has its shortage in the study of acupuncture as it may influence somewhat the effect of acupuncture, anesthesia is also of advantage in this kind of study due to that it can exclude the psychological disturbance. In the past decades many experimental studies conducted on the anesthetized animals have been published in top journals, showing a popular acceptance of the acupuncture studies carried out on the anesthetized animals. In addition, in the present study the rats in all groups including experimental and control ones were anesthetized equally and we can easily find out the significant differences of the recorded data between the EA group and SGIR group.

It is well recognized that during MIR [Ca^2+^]_i_ is increased, even overloaded due to the disorder of calcium homeostasis modulators. The [Ca^2+^]_i_ enhancement can lead to [Ca^2+^]_i_ oscillations resulted from the increased release of Ca^2+^ from sarcoplasmic reticulum. The [Ca^2+^]_i_ and its oscillation enhancement during and after repolarization phase are known to be arrhythmia-related substrates [24]. When heart is undergone ischemia and reperfusion, the sympathetic nervous system is over-excited [25]. which increases the release of catecholamine and leads to an over-stimulation of cardiac β_1_-adrenoceptor (β_1_-AR). The sensitivity and activity of β-adrenoceptors (β-ARs) are also facilitated during MIR [26]. While β_1_-AR stimulation is known to cause the increase in [Ca^2+^]_i_, even [Ca^2+^]_i_ overload [27], so as to bring about arrhythmias [28]. Therefore, both overstimulation of β-ARs and enhancement of [Ca^2+^]_i_ are considered as the arrhythmogenic substrates. Ischemia preconditioning, i.e. several times of short ischemia, is well known to protect the myocardium from the injury induced by following severe MIR [29]. Repetitive pretreatment of the cardiac myocytes with β-ARs agonist [30], high concentration of Ca^2+^ and the opener of L-Ca^2+^ channel can separately produce the same cardioprotective effect as ischemia preconditioning does [31, 32]. It is interesting that the activity of the sympathetic nervous system was reported to be affected by somatic stimulation [33]. It was also shown that EA stimulation significantly enhanced sympathetic activity [34]. Thus, the repetitive EA pretreatment may also achieve the cardioprotective effects via stimulating sympathetic nervous system and β_1_-AR. Our previous study showed that pretreatment with EA protects the heart from ischemic injury via inducing the functional attenuation of cardiac β_1_-AR [35]. Thus, we hypothesized an involvement of cardiac [Ca^2+^]_i_ in the mediation of the anti-arrhythmic effect of EA pretreatment. Our data showed that SGIR-induced increase of arrhythmic score was significantly attenuated with repetitive EA pretreatment and SGIR-induced increase in both quiescent [Ca^2+^]_i_ concentration and the number of [Ca^2+^]_i_ oscillations was also reduced by EA pretreatment. The results indicate that EA pretreatment may exert an anti-arrhythmic effect via reducing the augmented [Ca^2+^]_i_ concentration and oscillations by SGIR.

**Figure 5:**
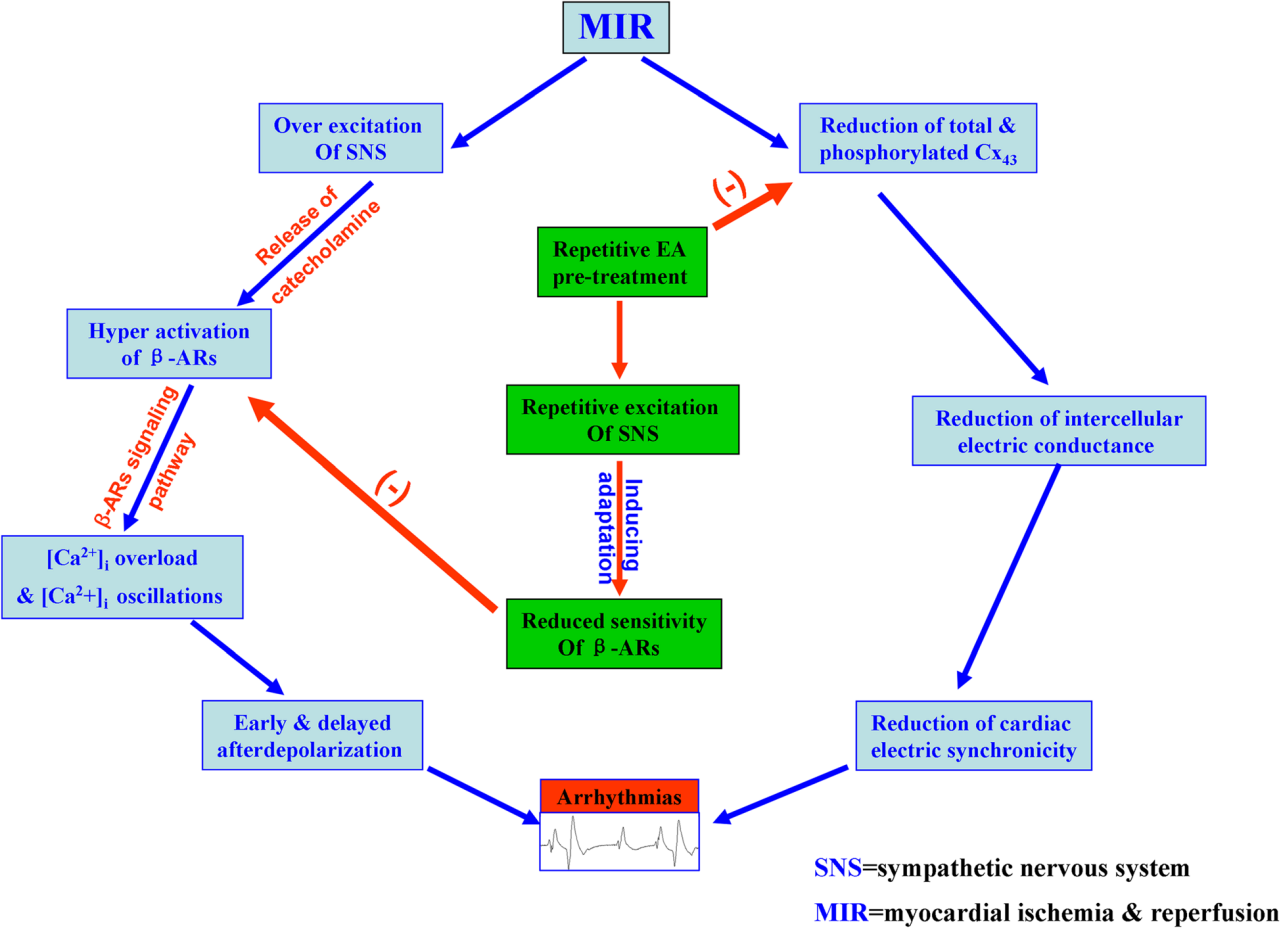
**Hypothetical diagram showing the possible targeted points mediating the anti-arrhythmic effect of EA pretreatment.** During MIR both sympathetic nervous system (SNS) and β-adrenoceptors (β-AR) were over excited, which leads to intracellular calcium ([Ca^2+^]_i_) overload and [Ca^2+^]_i_ oscillations; in addition, MIR also causes the reduction of total and phosphorylated Cx_43_, which subsequently attenuates the intercellular electric conductance and the cardiac electric synchronicity. Both the reduction of cardiac electric synchronicity and enhancement of [Ca^2+^]_i_ oscillations which is known to be related to early and delayed afterdepolarization can result in cardiac arrhythmias. On the one hand, repetitive electro-acupuncture (EA) pretreatment may finally produce the anti-arrhythmic effect via inducing an adaptation of β-AR to the stimulation of catecholamine released from the terminals of SNS, and bringing about the inhibition of both [Ca^2+^]_i_ overload and [Ca^2+^]_i_ oscillations; on the other hand, EA pretreatment may also inhibit the MIR-induced reduction of total or phosphorylated Cx_43_, which consequently enhances the cardiac electric synchronicity and then diminishes the occurrence of arrhythmias.

In brief, as shown in the hypothetical diagram of Figure 5, the possible mechanisms underlying the inhibition of calcium oscillations by EA pretreatment are as follows. Repetitive EA pretreatment may increase repetitively the activity of sympathetic nervous system (SNS) and the release of norepinephrine from the endings of SNS. The repetitively-enhanced norepinephrine then stimulates repetitively β_1_-AR and its down-stream signaling components, which may induce an adaptation or tolerance of the myocytes’ responsiveness to MIR which is known to increase [Ca^2+^]_i_ and induce [Ca^2+^]_i_ oscillations. Thus, the repetitive EA pretreatment-induced tolerance or adaptation to the [Ca^2+^]_i_ -enhancing MIR may finally reduce the [Ca^2+^]_i_ concentration and its oscillations as well.

Gap junctions, assembled with connexins, form cell-to-cell pathways for propagation of current flow, play an important role in the synchronous contraction of cardiac myocytes [36]. Cx_43_ is the predominant connexin expressed abundantly in ventricular and atrial myocytes. There are two different status of Cx_43_, namely, phosphorylated and non-phosphorylated ones. It is known so far that the phosphorylated Cx_43_ is a functionally active one, while the nonphosphorylated or dephosphorylated one is of no functional activity. Almost all the Cx_43_ are phosphorylated under normal condition, while during ischemia, Cx_43_ was progressively dephosphorylated without any changes in total content of Cx_43_ [37]. The alterations of Cx_43_ expression and phosphorylation status may induce serious arrhythmogenic substrate via attenuating the cell-to-cell coupling [38]. Furthermore, Cx_43_ was recently shown to play a role in mediating the cardioprotective effect of ischemic preconditioning [39], suggesting a possible involvement of Cx_43_ in the mediation of the anti-arrhythmic effect produced by repetitive EA pretreatment which mimics ischemia preconditioning. In the present study, although there was no significant difference in total content of Cx_43_ among NC, SGIR, EA and EAG groups, the level of nonphosphated Cx_43_ in the SGIR group was significantly higher than that in NC group (*P* <0.05). The increase in the nonphosphated Cx_43_ protein level was concomitant with the augmentation of [Ca^2+^]_i_ concentration, [Ca^2+^]_i_ oscillations and arrhythmia score. However, the nonphosphorylated Cx_43_ was significantly lower in EA group as compared with SGIR group, implying the improvement of the SGIR-impaired gap junction following the repetitive EA pretreatment. More interestingly, when the rats were treated with Cx_43_ antagonist 18-beta-glycyrrhetinic acid prior to the EA pretreatment, the EA pretreatment-induced attenuation of nonphosphorylated Cx_43_ was reversed, which supports the notion that Cx_43_ may be involved in the mediation of the anti-arrhythmic effect of EA pretreatment.

## Conclusions

In summary, EA could prevent cardiac arrhythmias induced by SGIR, this effect may be due, at least partly, to the inhibition on the Ca^2+^ overload, oscillations and the reduction of non-phosphorylated Cx_43_.

## Abbreviations

EA: Electro-acupuncture
[Ca^2+^]_i_: Intracellular Ca^2+^
MIR: Myocardial ischemia and reperfusion
Cx_43_: Connexin 43
SGIR: Simulative global ischemia and reperfusion
NC: Normal control
EAG: EA Plus 18 beta-glycyrrhetinic acid
VF: Ventricular fibrillation
VT: Ventricular tachycardia
PVC: Premature ventricular contraction
PMSF: Phenylmethylsulfonyl fluoride
DMSO: Diethyl sulphoxide
CHD: Coronary heart disease
β1-AR: β1-Adrenoceptor
β-ARs: β-Adrenoceptors
SNS: Sympathetic nervous system

## References

[1] Zipes DP, Wellens HJ (1998). Sudden cardiac death. Circulation.

[2] Lin JH, Shih CH, Kaphle K, Wu LS, Tseng WY, Chiu JH (2010). Acupuncture effects on cardiac functions measured by cardiac magnetic resonance imaging in a feline model. Evid Based Complement Alternat Med.

[3] Longhurst JC (2007). Electroacupuncture treatment of arrhythmias in myocardial ischemia. Am J Physiol Heart Circ Physiol.

[4] Lomuscio A, Belletti S, Battezzati PM, Lombardi F (2011). Efficacy of acupuncture in preventing atrial fibrillation recurrences after electrical cardioversion. J Cardiovasc Electrophysiol.

[5] Guo XQ, Jai RJ, Cao QY, Guo ZD, Li P (1981). Inhibitory effect of somatic nerve afferent impulses on the extrasystole induced by hypothalamic stimulation. Acta Physiol Sin.

[6] Guo XQ, Xia Y, Li P (1984). Role of arcuatus area in inhibitory action of somatic nerve stimulation on ventricular extrasystoles induced by hypothalamic stimulation in rabbits. Acta Physiol Sin.

[7] Lujan HL, Kramer VJ, DiCarlo SE (2007). Electroacupuncture decreases the susceptibility to ventricular tachycardia in conscious rats by reducing cardiac metabolic demand. Am J Physiol Heart Circ Physiol.

[8] Gao J, Fu W, Jin Z, Yu X (2006). A preliminary study on the cardioprotection of acupuncture pretreatment in rats with ischemia and reperfusion: involvement of cardiac beta-adrenoceptors. J Physiol Sci.

[9] Thimm J, Mechler A, Lin H, Rhee S, Lal R (2005). Calcium dependent open-closed conformations and interfacial energy maps of reconstituted hemichannels. J Biol Chem.

[10] Oyamada M, Tsujii E, Tanaka H, Matsushita T, Takamatsu T (2001). Abnormalities in gap junctions and Ca2+ dynamics in cardiomyocytes at the border zone of myocardial infarcts. Cell Commun Adhes.

[11] Lakatta EG, Talo A, Capogrossi MC, Spurgeon HA, Stern MD (1992). Spontaneous sarcoplasmic reticulum Ca2+ release leads to heterogeneity of contractile and electrical properties of the heart. Basic Res Cardiol.

[12] Yano M (2008). Ryanodine receptor as a new therapeutic target of heart failure and lethal arrhythmia. Circ J.

[13] Ladilov YV, Balser C, Piper HM (1997). Halothane protects cardiomyocytes against reoxygenation-induced hypercontracture. Circulation.

[14] Lameris TW, De Zeeuw S, Alberts G, Boomsma F, Duncker DJ, Verdouw PD (2000). Time course and mechanism of myocardial catecholamine release during transient ischemia in vivo. Circulation.

[15] Xia Q, Sheng JZ, Tai KK, Wong TM (1994). Effects of chronic U50, 488H treatment on binding and mechanical responses of the rat heats. J Pharmacol Exp Ther.

[16] Fenton RA, Galeckas KJ, Dobson JG (1995). Endogenous adenosine reduceds depression of cardiac function induced by beta-adrenergic stimulation during low flow perfusion. J Mol Cell Cardiol.

[17] Curtis MJ, Walker MJ (1988). Quantification of arrhythmias using scoring system: an examination of seven scores in an in vivo model of regional myocardial ischaemia. Cardiovasc Res.

[18] Dong H, Sheng JZ, Lee CM, Wong TM (1993). Calcium antagonistic and anti-arrhythmic actions of CPU-23, a substituted tetrahydroisoquinoline. Brit J Pharmacol.

[19] Yu XC, Li HY, Wang HX, Wong TM (1998). U50, 488H inhibits effects of norepinephrine in rat cardiomyocytes: cross talk between κ-opioid and β-adrenergic receptors. J Mol Cell Cardiol.

[20] Mackenzie L, Bootman MD, Laine M, Berridge MJ, Thuring J, Holmes A (2002). The role of inositol 1,4,5-trisphosphate receptors in Ca2+ signalling and the generation of arrhythmias in rat atrial myocytes. J Physiol.

[21] Yoshida K, Inui M, Saido TC, Sorimachi Y, Ishihara T, Kawashima S (1995). Reperfusion of rat heart after brief ischemia induces proteolysis of calspectrin (nonerythroid spectrin or fodrin) by calpain. Circ Res.

[22] Tsou MT, Huang CH, Chiu JH (2004). Electroacupuncture on PC6 (Neiguan) attenuates ischemia/reperfusion injury in rat hearts. Am J Chin Med.

[23] Cao Q, Liu J, Chen S, Han Z (1998). Effects of electroacupuncture at neiguan on myocardial microcirculation in rabbits with acute myocardial ischemia. J Tradit Chin Med.

[24] Kannankeril PJ, Mitchell BM, Goonasekera SA, Chelu MG, Zhang W, Sood S (2006). Mice with the R176Q cardiac ryanodine receptor mutation exhibit catecholamine-induced ventricular tachycardia and cardiomyopathy. Proc Natl Acad Sci U S A.

[25] Boachie-Ansah G, Kane KA, Parratt JR (1993). Is adenosine an endogenous myocardial protective (anti-arrhythmic) substance under conditions of ischaemia?. Cardiovasc Res.

[26] Chen H, Zhang YC, Li D, Phillips MI, Mehta P, Shi M (2000). Protection against myocardial dysfunction induced by global ischemia-reperfusion by antisense-oligodeoxynucleotides directed at beta(1)-adrenoceptor mRNA. J Pharmacol Exp Ther.

[27] Wang X, Wang J, Takeda S, Elimban V, Dhalla NS (2002). Alterations of cardiac beta-adrenoceptor mechanisms due to calcium depletion and repletion. Mol Cell Biochem.

[28] Thandroyen FT, Morris AC, Hagler HK, Ziman B, Pai L, Willerson JT (1991). Intracellular calcium transients and arrhythmia in isolated heart cells. Circ Res.

[29] Murry CE, Jennings RB, Reimer KA (1986). Preconditioning with ischemia: a delay of lethal cell injury in ischemic myocardium. Circulation.

[30] Frances C, Nazeyrollas P, Prevost A, Moreau F, Pisani J, Davani S (2003). Role of beta1- and beta2-Adrenoceptor Subtypes in Preconditioning Against Myocardial Dysfunction after Ischemia and Reperfusion. J Cardiovasc Pharmacol.

[31] Cain BS, Meldrum DR, Meng X, Shames BD, Banerjee A, Harken AH (1998). Calcium preconditioning in human myocardium. Ann Thorac Surg.

[32] Miyawaki H, Ashraf M (1997). Isoproterenol mimics calcium preconditioning-induced protection against ischemia. Am J Physiol Heart Circ Physiol.

[33] Sato A, Schmidt RF (1971). Spinal and supraspinal components of the reflex discharges into lumbar and thoracic white rami. J Physiol.

[34] Lin TB, Fu TC, Chen CF, Lin YJ, Chien CT (1998). Low and high frequency electroacupuncture at Hoku elicits a distinct mechanism to activate sympathetic nervous system in anesthetized rats. Neurosci Lett.

[35] Gao J, Fu W, Jin Z, Yu X (2007). Acupuncture pretreatment protects heart from injury in rats with myocardial ischemia and reperfusion via inhibition of the β1-adrenoceptor signalling pathway. Life Sci.

[36] Kimura H, Oyamada Y, Ohshika H, Mori M, Oyamada M (1995). Reversible inhibition of gap junctional intercellular communication, synchronous contraction, and synchronism of intracellular Ca2+ fluctuation in cultured neonatal rat cardiac myocytes by heptanol. Exp Cell Res.

[37] Beardslee MA, Lerner DL, Tadros PN, Laing JG, Beyer EC, Yamada KA (2000). Dephosphorylation and intracellular redistribution of ventricular connexin43 during electrical uncoupling induced by ischemia. Circ Res.

[38] Sato T, Ohkusa T, Honjo H, Suzuki S, Yoshida MA, Ishiguro YS (2008). Altered expression of connexin43 contributes to the arrhythmogenic substrate during the development of heart failure in cardiomyopathic hamster. Am J Physiol Heart Circ Physiol.

[39] Boengler K, Dodoni G, Rodriguez-Sinovas A, Cabestrero A, Ruiz-Meana R, Gres P (2005). Connexin 43 in cardiomyocyte mitochondria and its increase by ischemic preconditioning. Cardiovasc Res.

